# Measurement of Non-Stationary Characteristics of a Landfall Typhoon at the Jiangyin Bridge Site

**DOI:** 10.3390/s17102186

**Published:** 2017-09-22

**Authors:** Xuhui He, Hongxi Qin, Tianyou Tao, Wenshuo Liu, Hao Wang

**Affiliations:** 1 School of Civil Engineering, Central South University, Changsha 410006, China; xuhuihe@csu.edu.cn (X.H.); qinhongxi@csu.edu.cn (H.Q.); 2 School of Civil Engineering, Southeast University, Nanjing 211189, China; wanghao1980@seu.edu.cn

**Keywords:** wind characteristics, tropical storm, non-stationarity, field measurement, long-span suspension bridge

## Abstract

The wind-sensitive long-span suspension bridge is a vital element in land transportation. Understanding the wind characteristics at the bridge site is thus of great significance to the wind- resistant analysis of such a flexible structure. In this study, a strong wind event from a landfall typhoon called Soudelor recorded at the Jiangyin Bridge site with the anemometer is taken as the research object. As inherent time-varying trends are frequently captured in typhoon events, the wind characteristics of Soudelor are analyzed in a non-stationary perspective. The time-varying mean is first extracted with the wavelet-based self-adaptive method. Then, the non-stationary turbulent wind characteristics, e.g.; turbulence intensity, gust factor, turbulence integral scale, and power spectral density, are investigated and compared with the results from the stationary analysis. The comparison highlights the importance of non-stationary considerations of typhoon events, and a transition from stationarity to non-stationarity for the analysis of wind effects. The analytical results could help enrich the database of non-stationary wind characteristics, and are expected to provide references for the wind-resistant analysis of engineering structures in similar areas.

## 1. Introduction

Nowadays, the suspension bridge has become a preferable choice to cross rivers, valleys, or even seas in the land transportation. Many remarkable long-span suspension bridges have been constructed across the world, such as the Akashi-Kaikyo Bridge in Japan, the Xihoumen Bridge in China, and the Great Belt Bridge in Denmark [1]. This kind of bridge is distinguished by its superior spanning capability due to its inherent mechanical features. However, long-span suspension bridges are flexible systems sensitive to wind actions, and the sensitivity increases with the augment of the span length [2,3,4]. Thus, wind actions on long-span suspension bridges attract intensive attentions in engineering communities, and high requirements are put forward to guarantee the safety and serviceability of structures facing strong wind events, especially for those severe wind disasters (e.g., typhoon/hurricane, downburst, tornado). Improving the knowledge of strong wind cases is therefore of great significance to the security of long-span suspension bridges and similar structures.

In the past few decades, the characteristics of boundary layer winds have always been the research focus in structural wind engineering [3,5]. As more and more damages to property caused by typhoon events are frequently reported by public media, special attention is paid to the characteristics of this severe disaster [6,7,8]. A typhoon is an extreme wind event that frequently make destructive damages to engineering structures. For the wind-sensitive long-span suspension bridge, the fluctuations of typhoon winds are critical on the wind effects, emphasizing the demand to accurately analyze and accumulate the wind characteristics of typhoons.

The typical method to analyze the wind effects on a long-span suspension bridge is the Alan G. Davenport Wind Loading Chain [9,10], in which the underlying assumption is that the wind speed is stationary. In such a case, the fluctuations over a given time duration of a wind record is assumed to be a zero-mean stationary random process by subtracting a constant mean from the wind data. Accordingly, considerable results of wind characteristics obtained from field measurements have been widely adopted by specifications in different countries to guide the wind-resistant design of engineering structures [11,12,13]. However, strong non-stationary features are frequently captured in recent field measurements of typhoon events [14,15,16]. Significant time-dependent changes in the mean value, variance, frequency components, or their combinations are revealed as the inherent non-stationarities of typhoon winds [16], which are different from the stationary boundary layer wind. Hence, there will be a big barrier defending the utilization of the traditional stationary theory in the analysis of non-stationary wind records.

In order to solve the faced problem, a non-stationary wind model was proposed to characterize the features of typhoon winds and other extreme wind events [14,17]. In this model, the wind speed is treated as the superposition of a time-varying mean and a stationary turbulent component from a non-stationary perspective. Then, some pioneering work on the non-stationary wind characteristics of typhoon events is conducted. For example, Xu and Chen [14] extracted the time-varying trends of non-stationary wind records with the empirical mode decomposition and defined the non-stationary wind characteristics in a statistical view. Tao et al. [18] proposed a self-adaptive method to determine the time-varying mean and provided an exhaustive analysis on the non-stationary wind characteristics of a landfall typhoon. The non-stationary wind characteristics offer the parameters to simulate a more real wind environment of typhoons to guide the wind tunnel test or computer-aided simulation. Thus, a more precise buffeting analysis of such a long-span bridge can be conducted with non-stationary parameters included. Of course, the non-stationary consideration is expected to provide a more accurate result, which is greatly related to the safety and serviceability of long-span suspension bridges. Although some research on the non-stationary wind characteristics of typhoon winds has been carried out, the accumulated data is still insufficient to establish a database of non-stationary typhoon wind characteristics that could guide structural wind-resistant analysis and design. Also, the wind characteristics are heavily dependent on the geographical location, topography, and local terrains [1,3,7]. Hence, it is of great significance to investigate and accumulate the non-stationary wind characteristics during typhoons to further enrich the database for design.

In this study, a strong wind event from a landfall typhoon called Soudelor recorded by the anemometer on Jiangyin Bridge is taken for analysis. Both stationary and non-stationary gust values are characterized after the extraction of the time-varying mean with a wavelet-based method. Based on both stationary and non-stationary models, the wind characteristics, including turbulence intensity, gust factor, turbulence integral scale, and power spectral density, are then comprehensively analyzed with a comparison to the recommendations by current specifications [11,12]. The comparison highlights the importance of non-stationary considerations of typhoon events. The analytical results can help enrich the database of non-stationary wind characteristics and are expected to provide references for the wind-resistant analysis of engineering structures in similar areas.

## 2. Measurement and Data Source

### 2.1. Anemometer Utilized in Field Measurement

**Figure 1:**
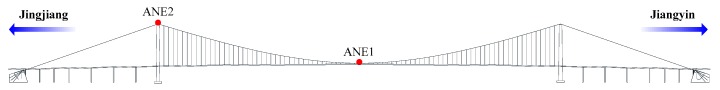
Layout of anemometers on Jiangyin Bridge.

Two anemometers are installed in the structural health monitoring systems of Jiangyin Bridge for the field measurement. As shown in Figure 1, one anemometer is installed in the middle of the main span (53.0 m above the ground), while the other is on top of the northern tower (197 m above the ground). The anemometer is an HD2003.1-type three-axis ultra-sonic anemometer made by the Delta OHM Company in Padova, Italy. The wind speed ranging from 0 m/s to 60 m/s can be accurately recorded with a resolution of 0.01 m/s, while the wind direction in the range between 0° and 359.9° can be measured with a resolution of 0.1°. The upper limit of the sampling frequency of the anemometer can reach 50 Hz. For the convenient storage of the massive data, the sampling frequency during measurements is set as 1 Hz. During the measurement, the two dimensional mode is activated for the anemometer, so only the wind speed and direction are recorded. In respect to the wind direction, due north is defined as 0° with a positive direction rotating clockwise.

### 2.2. Description of Typhoon Soudelor

**Figure 2:**
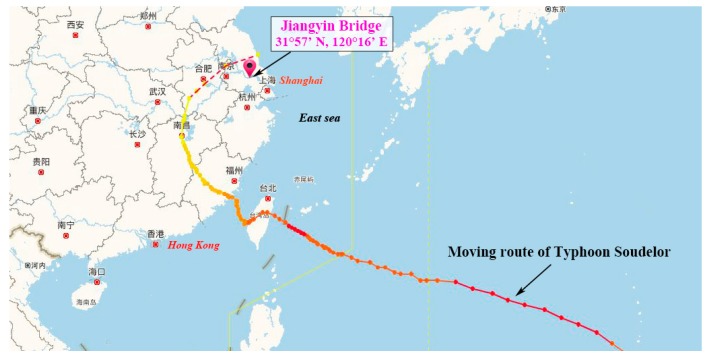
Moving route of Typhoon Soudelor.

Typhoon Soudelor was the 13th tropical cyclone in 2015. It was born as a tropical storm on the northwest surface of the Pacific Ocean and moved towards northwest on 30th July. On 3rd August, the intensity of Soudelor was further developed and achieved the same level of a super typhoon. The maximum wind speed near sea surface could achieve 65 m/s. On the morning of 8th August, it made landfall in Taiwan and then entered the mainland of China from Fujian Province. Afterwards, it downgraded as a tropical storm and passed through Jiangxi Province and Anhui Province. The whole moving route of Typhoon Soudelor is shown in Figure 2. As there is an about 500 km distance between Jiangyin Bridge site and the nearest moving route of Soudelor, the strong wind data was collected at the outer vortex region.

## 3. Mean Wind Characteristics

### 3.1. Measured Wind Samples of Typhoon Soudelor

**Figure 3:**
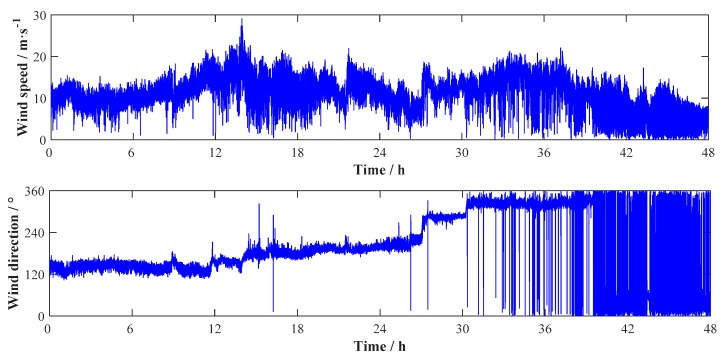
Measured wind samples during Typhoon Soudelor.

During the strong wind event, the anemometers on Jiangyin Bridge successfully recorded the wind speeds and directions. Among the measurements, the wind record from 00:00:00 on 10th August to 23:59:59 on 11th August is selected for analysis. The full wind record collected by the anemometer ANE2 on top of the tower is shown in Figure 3. The high oscillation of the wind direction from 30 h to 48 h is due to the wind direction of 360° equaling to 0°. The wind record in Figure 3 will be utilized for the following analyses.

**Figure 4:**
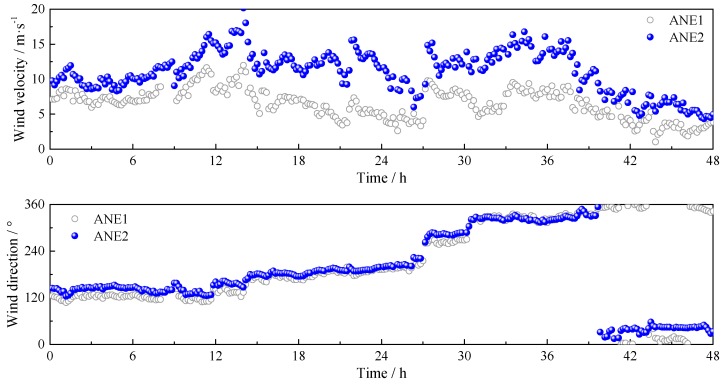
Comparison of the wind samples from ANE1 and ANE2.

In order to validate the quality of the field measured data in Figure 3, the simultaneously collected wind samples by ANE1 are selected for comparison. The wind speed and direction in 10 min intervals of ANE1 and ANE2 are presented in Figure 4. The mean wind speed and direction are calculated according to the vector decomposition method [3]. The variation tendencies of the mean wind speeds are similar, and the mean wind directions coincide well with each other, which indicate that the wind data utilized for the following analyses is reliable.

### 3.2. General Wind Models

In traditional analysis of the wind data, the wind speed is assumed to be an ergodic stationary random process consisting of a constant mean and a zero-mean turbulence, which is detailed as

(1) $$U(t)=\bar{U}+u(t)$$

where U(t) is the wind speed; $\bar{U}$ is the constant mean over a time interval *T*, which is chosen as 10 min in Chinese code [12]; and u(t) is the fluctuating component.

In the non-stationary wind model, a deterministic time-varying trend is captured in the wind speed. Hence, the wind speed is treaded as the superposition of a time-varying mean and a residual zero-mean stationary random process, as presented in Equation (2).

(2) $$U(t)={\tilde{U}}^{*}(t)+u^{*}(t)$$

where ${\tilde{U}}^{*}(t)$ is the time-varying mean reflecting the temporal trend of wind speed; and $u^{*}(t)$ is the fluctuating wind in the non-stationary model. Obviously, if the wind speed is a stationary process, the time-varying mean in Equation (2) will be reduced to a constant mean, which is the same as that of Equation (1), so that Equation (2) will be the same with Equation (1). Therefore, Equation (2) is a more general form for the analysis of the wind data. It should be noted that the models presented by Equations (1) and (2) are adapted to both the longitudinal and lateral cases, but the mean value of the lateral turbulence equals zero.

### 3.3. Extraction of the Time-Varying Mean

When utilizing the non-stationary wind model, a critical step is to detrend the time-varying mean from the wind data. Many methods have been developed to extract the temporal trend for non-stationary wind records, among which empirical mode decomposition (EMD) and wavelet transform (WT) are two popular and frequently used approaches. For example, Chen and Letchford [18] estimated the time-varying mean for downburst wind by employing db4 wavelet. Wang et al. [19] selected db20 wavelet to acquire the temporal mean of a downburst data. Xu and Chen [14] extracted the time-varying mean of a strong wind via EMD. However, the selection of decomposition levels depends on users’ judgment, which puts forward a requirement for a straightforward treatment of this procedure. Su et al. [20] proposed a scheme to derive a reasonable time-varying mean, but the procedure is relatively complicated since the estimation of EPSD and structural response computation is included. Tao et al. [21] developed a self-adaptive WT-based approach to extract the time-varying mean according the stationarity of the signal.

**Figure 5:**
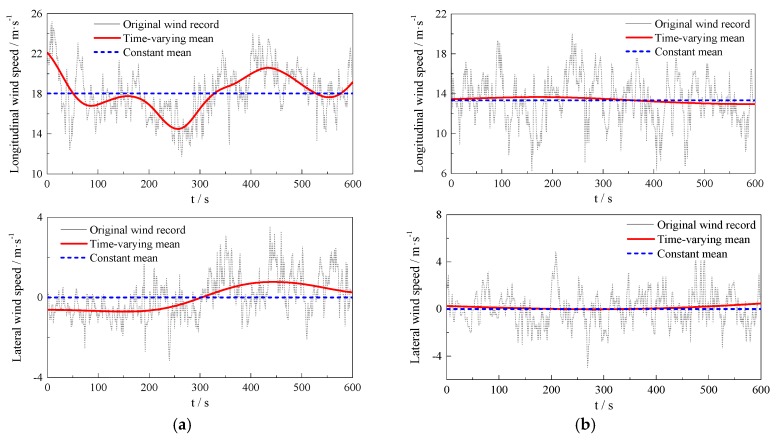
Time-varying mean wind speed versus constant mean wind speed of wind records: (**a**) non-stationary records; (**b**) stationary records.

With the wavelet-based self-adaptive method by Tao et al. [21], the time-varying trends of the wind records are separated for Typhoon Soudelor with the db20 wavelet. In this study, the time interval utilized is selected as 10 min according to the Chinese specification [12]. Some of the typical samples including stationary and non-stationary records are shown in Figure 5. As illustrated in Figure 5, the time-varying mean differs much from the constant mean for the non-stationary wind records, which means the stationary assumption cannot be utilized anymore. For the stationary records, the extracted time-varying mean is almost the same as the constant mean, indicating the unification of Equations (1) and (2) for stationary records. The performance of stationary and non-stationary records verifies the effectiveness of the self-adaptive method. Comparing the differences between the time-varying mean and constant mean in non-stationary longitudinal and lateral turbulences, the longitudinal wind speed presents a stronger non-stationarity than the other case.

## 4. Turbulent Wind Characteristics

### 4.1. Turbulence Intensity

The turbulence intensity in the traditional stationary theory is defined as the ratio of standard deviation of turbulence in each direction to the constant mean wind speed in a given time interval, as expressed in Equation (3). The non-stationary turbulence intensity is defined as Equation (4), making it have the same physical meaning with that of the stationary model.

(3) $$I_{i}=\frac{\sigma_{i}}{\bar{U}},\;i=u,v$$

(4) $$I_{i}^{*}=E{\left[\frac{\sigma_{i}^{*}}{\tilde{U}(t)}\right]}_{T},i=u,v$$

where $I_{i}$ and $I_{i}^{*}$ are stationary and non-stationary turbulence intensities, respectively; u and v denote the cases of longitudinal and lateral turbulences; $\sigma_{i}$ and $\sigma_{i}^{*}$ are standard deviations of turbulences in stationary and non-stationary wind models, respectively; and E[·] represents the mean value over the time interval *T*, which is set as 10 min in this study.

**Figure 6:**
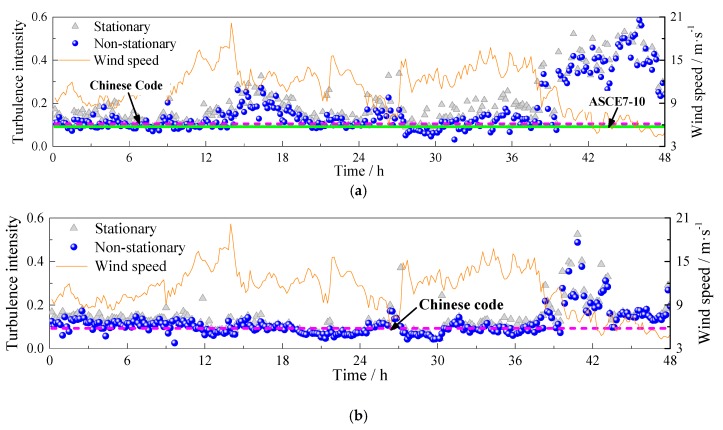
Comparison of stationary and non-stationary turbulence intensities: (**a**) longitudinal case; (**b**) lateral case.

Based on the stationary and non-stationary models expressed by Equations (3) and (4), the turbulence intensities of Typhoon Soudelor are calculated from stationary and non-stationary perspectives. The results are presented in Figure 6. It is noteworthy that the non-stationary turbulence intensities are smaller than stationary ones for both longitudinal and lateral cases. The difference in the longitudinal case is more obvious than that in the lateral case, which means the non-stationarity of the longitudinal turbulence is much stronger than that of the lateral wind.

The longitudinal turbulence intensity profile for categories B, C, and D exposure in ASCE7-10 is given by [11]:

(5) $$I_{u}=c{\left(\frac{10}{z}\right)}^{1/6}$$

where *c* equals to 0.15 for category D exposure. Thus, the longitudinal turbulence intensity at an altitude of 197 m is suggested as 0.091 by ASCE7-10 [11]. In Chinese code [12], a ratio between longitudinal and lateral turbulence intensities is suggested as 1:0.88, and the value for longitudinal case is recommended as 0.10 within the altitude between 150 m and 200 m.

The recommendations of turbulence intensities in ASCE7-10 and Chinese code are also plotted in Figure 6. It is shown in Figure 6 that the suggestions from both ASCE7-10 and Chinese code generally coincide well with the measured turbulence intensities from 0 h to 39 h for both longitudinal and lateral cases of Typhoon Soudelor. However, the turbulence intensities from 39 h to 48 h are much larger than those in the range 0–39 h. This phenomenon results from the low wind speed case after the passage of typhoon winds [3]. The mean wind speed is also plotted in Figure 6 to show the variation of wind speeds. The wind speed corresponds the right Y-axis of Figure 6. For longitudinal turbulence intensities during 14–20 h, they are a little larger than those in other durations from 0 h to 39 h and higher than recommendations from both ASCE7-10 and Chinese code. This is mainly attributed to the significant convective properties of typhoon events; the turbulence intensity is thus not homogenous in the entire duration.

### 4.2. Gust Factor

The gust factor is an important parameter that can convert the mean wind speed to maximum gust value. In the stationary model, it is defined as the ratio of the peak mean wind speed in a gust duration *t_g_* to the mean wind speed over the time interval *T* [11,12], which is expressed as Equation (6). For the non-stationary gust factor, it is defined as the maximum ratio of the averaged original wind speed to averaged time-varying mean within a given time interval *T*, among which both the original wind speed and time-varying mean are averaged in the gust duration *t_g_* [14,21].

(6) $$G_{u}(t_{g},T)=\frac{\max{\left[U(t_{g})\right]}_{T}}{\bar{U}}$$

(7) $$G_{u}^{*}(t_{g},T)=\max{\left[\frac{U(t_{g})}{{\tilde{U}}^{*}(t_{g})}\right]}_{T}$$

where $G_{u}(t_{g},T)$ and $G_{u}^{*}(t_{g},T)$ are stationary and non-stationary gust factors, respectively; $U(t_{g})$ is the mean wind speed over a gust duration *t_g_*; and ${\tilde{U}}^{*}(t_{g})$ is the mean value of the time-varying mean over the duration *t_g_*. In this study, the gust duration is selected as 3 s according to ASCE7-10.

**Figure 7:**
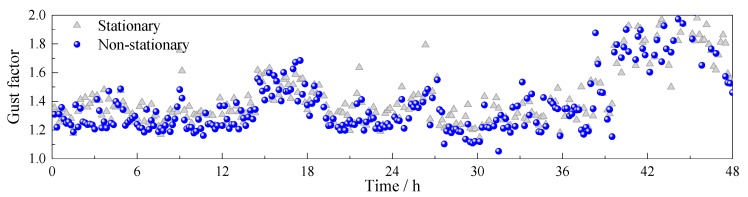
Comparison of stationary and non-stationary gust factor.

A comparison of stationary and non-stationary gust factors of the wind records is presented in Figure 7. Similar to the phenomenon that captured in the turbulence intensity, the non-stationary gust factor is generally lower than the stationary case. The variation trend of the gust factor along the time axis is similar to that of the longitudinal turbulence intensity; thus, there may exist a strong relationship between the gust factor and the longitudinal turbulence intensity.

**Figure 8:**
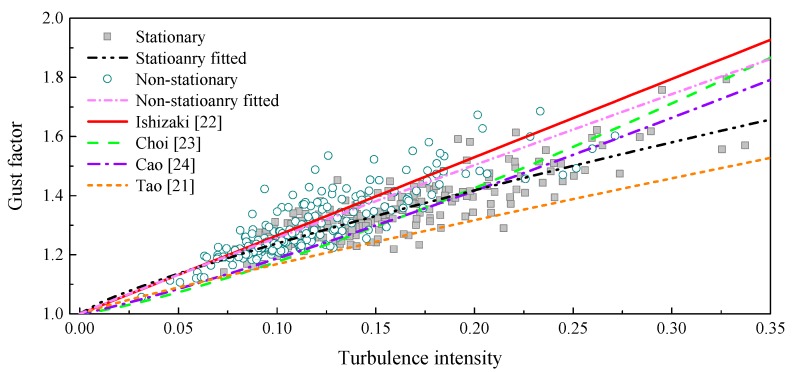
Stationary and non-stationary gust factors versus the turbulence intensity.

With an analysis of the definition of the gust factor, it is found to be strongly related to the longitudinal turbulence intensity, since the mean wind speed in a gust duration is positively correlated with the standard deviation of turbulence. The stationary and non-stationary gust factors versus the corresponding turbulence intensities are plotted in Figure 8, in which the turbulence intensities of the aforementioned low wind speed cases are not included. It is easy to find that there is a positive relationship between the gust factor and the turbulence intensity. The presented relationships of stationary and non-stationary cases are similar to each other.

Some expressions of the relationship between gust factor and the longitudinal turbulence intensity have been proposed based on linear or nonlinear models, which can be expressed in the general form below,

(8) $$G_{u}(t_{g},T)=1+k_{1}I_{u}^{k_{2}}\ln\left(\frac{T}{t_{g}}\right)$$

where Ishizaki suggested *k*_1_ = 0.5, *k*_2_ = 1.0 for typhoons [22]; Choi suggested *k*_1_ = 0.62, *k*_2_ = 1.27 [23], Cao suggested *k*_1_ = 0.5, *k*_2_ = 1.15 for Typhoon Maemi [24], and Tao et al.; suggested *k*_1_ = 0.22, *k*_2_ = 0.84 for a non-stationary case.

The relationships of stationary and non-stationary cases between gust factor and turbulence intensity are also fitted for comparison, where the stationary case suggests *k*_1_ = 0.29, *k*_2_ = 0.81 and the non-stationary case suggests *k*_1_ = 0.45, *k*_2_ = 0.96. The R^2^ values of the fitting for stationary and non-stationary cases are 0.85 and 0.80, respectively. The fitted expressions together with the empirical models are also plotted in Figure 8. As shown in Figure 8, the two fitted models can well describe the stationary and non-stationary relationships between gust factor and turbulence intensity. Although the Ishizaki model was proposed for the stationary wind model, it is similar to the fitted non-stationary expression in this study and can well describe the measured relationship between non-stationary gust factor and non-stationary turbulence intensity. For the stationary relationship, only the Tao model in empirical expressions provides a similar trend but it behaves as the lower limit of the measured cases.

### 4.3. Turbulence Integral Scale

Turbulence integral scale (TIS) is a measure of the average size of turbulent eddies [1]. In the stationary theory, TIS is mathematically defined as Equation (9) with the preliminary Taylor hypothesis. With the physical meaning unchanged, the non-stationary TIS is defined from a statistical view, as expressed in Equation (10).

(9) $$L_{i}=\frac{\bar{U}}{\sigma_{i}^{2}}\int_{0}^{\infty }R_{i}(\tau )d\tau \;i=u,v$$

(10) $$L_{i}^{*}=E\left[\frac{\tilde{U}(t)}{{\left(\sigma_{i}^{*}\right)}^{2}}\int_{0}^{\infty }R_{i}^{*}(\tau )d\tau \right]\;i=u,v$$

where $L_{i}$ and $L_{i}^{*}$ are stationary and non-stationary turbulence integral scales, respectively; $R_{i}(\tau )$ and $R_{i}^{*}(\tau )$ are auto-covariance functions of the stationary and non-stationary fluctuations, respectively; and τ is the lag time. In order to avoid the errors induced by the estimated fluctuations of covariance functions, the upper limit of the integral is suggested to take the first *t* where $R_{i}(\tau )=0.05\sigma_{i}^{2}$ and $R_{i}^{*}(\tau )=0.05{\left(\sigma_{i}^{*}\right)}^{2}$.

Stationary and non-stationary TIS in longitudinal and lateral cases of Typhoon Soudelor are calculated according to Equations (9) and (10). The corresponding results are presented in Figure 8. It can be seen that non-stationary turbulence integral scales are totally smaller than stationary ones, which is mainly attributed to the extraction of the time-varying mean. Detrending the non-stationary wind records will filter some low-frequency components, which reflect the large-scale eddies in turbulence.

**Figure 9:**
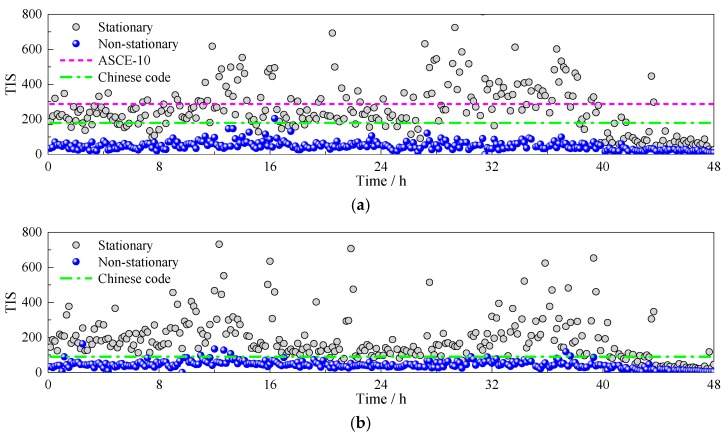
Turbulence integral scales based on stationary and non-stationary wind models: (**a**) longitudinal case; (**b**) lateral case.

The longitudinal turbulence integral scale profile for categories B, C, and D exposure in ASCE7-10 is given by [11]

(11) $$L_{u}=l{\left(\frac{z}{10}\right)}^{\varepsilon }$$

where *l* is equals to 198.2 and *ε* is equals to 1/8 for category D exposure. At the height of 197 m, the longitudinal turbulence integral scale is recommended as 287.68 by ASCE7-10. In Chinese specification, longitudinal and lateral turbulence integral scales between the height of 150 m and 200 m are suggested as 180 m and 90 m, respectively. The turbulence integral scales recommended by ASCE7-10 and Chinese code are also plotted in Figure 9.

As shown in Figure 9, ASCE7-10 provides a good estimate for the stationary longitudinal turbulence integral scale of Typhoon Soudelor. The mean value of measured turbulence integral scales is 261.8 m, which is with a 9.0% deviation from the suggested value by ASCE7-10. However, the recommended values by Chinese code are generally lower than measured TIS for both longitudinal and lateral cases. The mean stationary TIS of lateral turbulence is 206.8, so the ratio between longitudinal and lateral TIS is 1:0.79, which means the lateral TIS is underestimated by Chinese code. For the non-stationary TIS, the mean values of longitudinal and lateral cases are 49.4 and 44.4, respectively. Thus the ratio between the two cases is 1:0.90, which is similar to the results acquired by Tao et al. [21]. This means non-stationary longitudinal and lateral turbulence integral scales are close to each other, indicating the average size of turbulent eddies is similar in the two directions by the non-stationary wind model.

### 4.4. Turbulence Power Spectral Density

Turbulence power spectral density, which characterizes the energy distribution of turbulence in frequency domain, is a critical element in the precise prediction of buffeting responses of engineering structures. Based on the stationary assumption, many spectral models have been proposed via the field measured data in strong wind events. For example, Kaimal spectrum [25] is widely accepted for longitudinal turbulence and employed in the wind resistant-design specification for highway bridges in China [12]. The expression of Kaimal spectrum is detailed as:

(12) $$\frac{nS_{u}(n)}{{\left(u_{*}\right)}^{2}}=\frac{200f}{{\left(1+50f\right)}^{5/3}}$$

where $S_{u}(n)$ is the turbulence power spectral density; n is the natural frequency of turbulence; $u_{*}$ is the friction wind speed, which can be approximated by $\sigma_{u}^{2}/6$; and f is the Monin coordinate and equals to $nz/\bar{U}$, in which z is the altitude of the wind speed.

For the non-stationary power spectral density, the following form is suggested with the physical meaning unchanged.

(13) $$\frac{n{\tilde{S}}_{u}(n)}{{\left({\tilde{u}}_{*}^{}\right)}^{2}}=\frac{200\tilde{f}}{{\left(1+50\tilde{f}\right)}^{5/3}}$$

where ${\tilde{S}}_{u}(n)$ is the non-stationary power spectral density; ${\tilde{u}}_{*}^{}$ is the non-stationary friction wind velocity and can be approximated as ${\left(\sigma_{u}^{*}\right)}^{2}/6$; and $\tilde{f}$ is the non-stationary Mornin coordinate and equals to $nz/E\left[\tilde{U}(t)\right]$.

**Figure 10:**
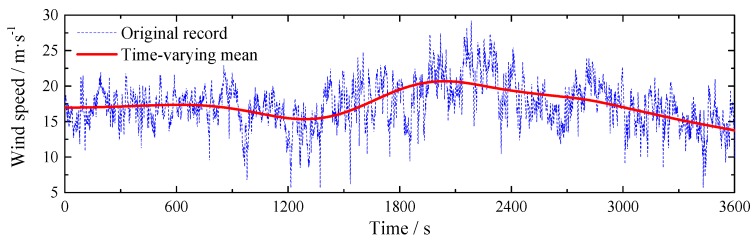
Wind record for power spectral density analysis.

For the spectral analysis, a strong wind sample, which includes the maximum wind speed, from 13.5 h to 14.5 h in Figure 3, is selected as an example with 1 h duration. The original wind speed of this record is shown in Figure 10. Meanwhile, the time-varying mean is also plotted for comparison. The mean wind speed of this sample is 17.41 m/s. The stationary turbulence intensity and turbulence integral scale are 0.183 and 1509.8 m, while the corresponding non-stationary values are 0.156 and 228.8 m.

**Figure 11:**
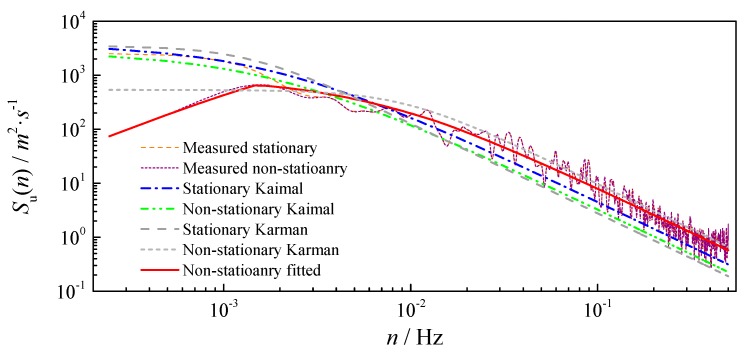
Comparison between measured power spectral densities and empirical models.

After subtracting the constant mean and time-varying mean from the original wind record separately, the stationary and non-stationary turbulences are obtained. Based on the Fourier transform, the stationary and non-stationary power spectral densities of turbulence are estimated, and the results are presented in Figure 11. Obviously, the non-stationary power spectral density is lower than the stationary case in low-frequency ranges, while the two cases keep consistent in the rest of the ranges. This phenomenon can be well explained by the filtering of the time-varying mean, which physically performs as the low-frequency content in wind fluctuations [1]. For a long-span suspension bridge like Jiangyin suspension bridge, the background or resonant buffeting responses are mainly dominated by low-frequency mode shapes. The lowest frequency of Jiangyin Bridge is about 0.05 Hz, so the non-stationarity of typhoon events may only influence the background buffeting responses. However, with the development of long-span suspension bridges, the structural lowest frequency will be much smaller and gradually approach the discrepancy of stationary and non-stationary turbulence captured in this study. Thus, the captured difference between stationary and non-stationary power spectral densities may greatly affect the accuracy of predicted buffeting responses.

In order to verify the effectiveness of the stationary and non-stationary empirical models, the stationary and non-stationary Kaimal spectra are also plotted in Figure 10 for comparisons. It is easy to find that neither of the stationary nor non-stationary Kaimal spectrum can well describe the measured spectra, including both stationary and non-stationary cases. They are lower than measured spectra in high-frequency ranges. The stationary Kaimal spectrum is able to describe the low-frequency feature of stationary power spectral density, but the non-stationary Kaimal spectrum cannot describe the descending feature of non-stationary case in the low-frequency range. Since the Von Karman spectrum is widely utilized in the building engineering, its stationary and non-stationary forms are also plotted in Figure 11. It is shown that the stationary Karman spectrum cannot well describe the measured power spectral density. The non-stationary Karman spectrum can capture most of the measured power spectral density, but the low-frequency feature of the non-stationary case is still not able to be presented. Hence, new models need to be utilized to perfectly describe the non-stationary power spectral density.

To cope with the faced problem, Tao et al. [1] proposed a general model for non-stationary turbulence via a modulating function in frequency domain. The non-stationary empirical model is suggested as

(14) $$S_{u}^{*}(n)=A(n)\times S_{u}^{}(n)$$

where $S_{u}^{*}(n)$ is the non-stationary power spectral density; A(n) is the frequency modulating function; and $S_{u}^{}(n)$ is the empirical model via nonlinear fitting. In this study, the benchmark model utilized for nonlinear fitting is selected as:

(15) $$\frac{nS_{u}(n)}{{\left(u^{*}\right)}^{2}}=\frac{Af}{{\left(B+Cf\right)}^{5/3}}$$

where *A*, *B*, *C* are constants to be determined.

Based on the nonlinear fitting to the measured non-stationary power spectral density, the following expression by Equation (16) can be obtained. Then, a modulating function is also calculated, and its detailed expression is presented in Equation (17).

(16) $$\frac{nS_{u}(n)}{{\left(u^{*}\right)}^{2}}=\frac{1.982f}{{\left(0.118+2.166f\right)}^{5/3}}$$

(17) $$A(n)=\{\begin{array}{cc} \exp(1.412\ln n+8.928) & 0\leq n\leq 0.0015\\ \exp(0.111\ln n+0.467) & 0.0015<n\leq 0.015\\ 1 & n>0.015 \end{array}$$

The obtained empirical non-stationary power spectral density is also plotted in Figure 10. It can be seen that the fitted non-stationary model can perfectly satisfy with the non-stationary power spectrum, indicating the effectiveness of the empirical model obtained after modulation. The general model by Tao et al. is also verified to be effective for non-stationary turbulence. Hence, it can be inferred that more accurate results will be acquired in the buffeting analysis of long-span suspension bridges if the general model is utilized.

It should be noted that the fitted spectrum is obtained with the presented 1 h long recording. Due to the distinct convective features existing in typhoon events, the non-stationary spectrum of other records may deviate from the presented fitted spectrum. Hence, the measurements still need to be accumulated to perfect the expression of the empirical model of non-stationary turbulence in typhoon winds.

## 5. Conclusions

In this study, the non-stationary wind characteristics of a landfall typhoon at the Jiangyin Bridge site is analyzed with a comparison of the results from stationary analysis. The following conclusions can be drawn accordingly.

- The time-varying mean differs much from the constant mean for the non-stationary wind records, which means the stationary assumption is invalid. For the longitudinal and lateral turbulences of Typhoon Soudelor, the longitudinal wind speed presents stronger non-stationarity than the other case.
- The non-stationary turbulence intensities are smaller than stationary ones for both longitudinal and lateral cases. The difference in the longitudinal case is more obvious than that in the lateral case, which means the non-stationarity of the longitudinal turbulence is much stronger than that of the lateral wind.
- Except low wind speed cases, the suggestions from both ASCE7-10 and Chinese code generally coincide well with the measured turbulence intensities for both longitudinal and lateral cases of Typhoon Soudelor.
- The two fitted models can well describe the stationary and non-stationary relationships between gust factor and turbulence intensity. Among the presented empirical models, only the Ishizaki model is similar to the fitted non-stationary expression, and can well describe the measured relationship between non-stationary gust factor and non-stationary turbulence intensity.
- Non-stationary turbulence integral scales are much smaller than stationary ones, which is mainly attributed to the extraction of the time-varying mean. Detrending the non-stationary wind records will filter some low-frequency components, which reflect the large-scale eddies in turbulence.
- The non-stationary power spectral density is lower than the stationary case in low-frequency ranges, while the two cases keep consistent in the rest of the ranges. This phenomenon can be well explained by the filtering of the time-varying mean, which physically performs as the low-frequency content in wind fluctuations.
- The fitted non-stationary model via modulation can perfectly satisfy the measured non-stationary power spectrum, indicating the effectiveness of the general model for non-stationary turbulence.
- There is a significant difference between stationary and non-stationary wind characteristics. In light of the typhoon cases with strong non-stationarity, a shift consideration from stationarity to non-stationarity is highlighted for the analysis of wind effects.

In general, a non-stationary model to be developed is recommended to guide the design or prediction of responses of long-span bridges in the future, since non-stationary features are frequently captured in extreme wind events and accurately simulating the buffeting responses is the main purpose of engineering practices. The challenges for this shift consideration are to include the non-stationary wind effects in the all the main components including forces, the bridge system, and responses during the simulation.
